# Acetyl-CoA carboxylase inhibitor increases LDL-apoB production rate in NASH with cirrhosis: prevention by fenofibrate

**DOI:** 10.1016/j.jlr.2023.100339

**Published:** 2023-02-02

**Authors:** Mohamad Dandan, Julia Han, Sabrina Mann, Rachael Kim, Kelvin Li, Hussein Mohammed, Jen-Chieh Chuang, Kaiyi Zhu, Andrew N. Billin, Ryan S. Huss, Chuhan Chung, Robert P. Myers, Marc Hellerstein

**Affiliations:** 1 Department of Nutritional Sciences and Toxicology, Graduate Program in Metabolic Biology, University of California at Berkeley, Berkeley, CA, USA; 2 Gilead Sciences, Inc, Foster City, CA, USA

**Keywords:** nonalcoholic steatohepatitis, LDL, lipoproteins, lipoproteins/kinetics, lipoproteins/metabolism, triglycerides, mass spectrometry, hypertriglyceridemia, firsocostat, fenofibrate, ACC, acetyl-CoA carboxylase, ACCi, ACC inhibitor, ASR, absolute synthesis rate, DNL, de novo lipogenesis, FRR, fractional replacement rate, MRE, magnetic resonance elastography, NAFLD, nonalcoholic fatty liver disease, NASH, nonalcoholic steatohepatitis, TG, triglyceride

## Abstract

Treatment with acetyl-CoA carboxylase inhibitors (ACCi) in nonalcoholic steatohepatitis (NASH) may increase plasma triglycerides (TGs), with variable changes in apoB concentrations. ACC is rate limiting in de novo lipogenesis and regulates fatty acid oxidation, making it an attractive therapeutic target in NASH. Our objectives were to determine the effects of the ACCi, firsocostat, on production rates of plasma LDL-apoB in NASH and the effects of combined therapy with fenofibrate. Metabolic labeling with heavy water and tandem mass spectrometric analysis of LDL-apoB enrichments was performed in 16 NASH patients treated with firsocostat for 12 weeks and in 29 NASH subjects treated with firsocostat and fenofibrate for 12 weeks. In NASH on firsocostat, plasma TG increased significantly by 17% from baseline to week 12 (*P* = 0.0056). Significant increases were also observed in LDL-apoB fractional replacement rate (baseline to week 12: 31 ± 20.2 to 46 ± 22.6%/day, *P* = 0.03) and absolute synthesis rate (ASR) (30.4–45.2 mg/dl/day, *P* = 0.016) but not plasma apoB concentrations. The effect of firsocostat on LDL-apoB ASR was restricted to patients with cirrhosis (21.0 ± 9.6 at baseline and 44.2 ± 17 mg/dl/day at week 12, *P* = 0.002, N = 8); noncirrhotic patients did not change (39.8 ± 20.8 and 46.3 ± 14.8 mg/dl/day, respectively, *P* = 0.51, N = 8). Combination treatment with fenofibrate and firsocostat prevented increases in plasma TG, LDL-apoB fractional replacement rate, and ASR. In summary, in NASH with cirrhosis, ACCi treatment increases LDL-apoB100 production rate and this effect can be prevented by concurrent fenofibrate therapy.

## Graphical abstract

Graphical abstract: ACCi treatment alone *(green arrow)* leads to increased apoB particle production in the liver as shown by increased production of LDL-apoB. Fenofibrate combined with ACCi treatment *(red inhibitory symbol)* prevented the increased LDL-apoB particle production.

**Figure:**
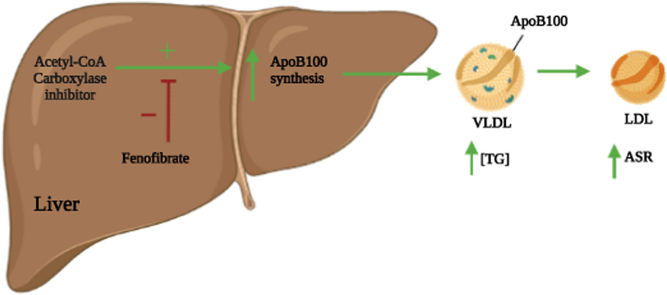

Over 90 million Americans have nonalcoholic fatty liver disease (NAFLD), a condition characterized by excessive liver fat and chronic inflammation (1, 2). The cause is unclear but it is associated with obesity, diabetes, and metabolic syndrome (3). In a subset of individuals with NAFLD, progression to nonalcoholic steatohepatitis (NASH), cirrhosis, and hepatocellular carcinoma may occur (3, 4). Dyslipidemias are common in NAFLD patients and are associated with increased risk of cardiovascular disease and progression to NASH (5, 6, 7). Hypertriglyceridemia is particularly common in NAFLD and can be influenced by pharmacological treatment (5, 8).

An attractive therapeutic target for NAFLD is inhibition of acetyl-CoA carboxylase (ACC), which catalyzes the rate limiting step of hepatic de novo lipogenesis (DNL) and regulates fatty acid oxidation (8, 9). Interestingly, observations in NASH patients in phase 2 clinical trials, ACC knockout mouse models, and preclinical models exhibited not only reductions in liver fat content but also hypertriglyceridemia (8, 10, 11, 12, 13, 14, 15, 16). The latter was unexpected, as reduced hepatic malonyl-CoA production by ACC inhibition was anticipated both to reduce synthesis and to increase oxidation of fatty acids in the liver (17). In addition, ACC inhibitors (ACCi) in NASH patients has been reported to increase apoB-containing lipoproteins as well as VLDL particle number, triglyceride (TG) content, and secretion (8, 10, 11, 12, 13, 14, 15, 16).

A key question is whether this effect of ACCi acts on the liver through increased production of apoB100-containing particles, or on tissue clearance of plasma lipids, or apoB-containing lipoproteins, as these may confer differences in atherogenicity and suggest different treatment approaches. Data from animal models have suggested that ACCi can cause both changes in hepatic lipid metabolism (8) and peripheral lipoprotein lipase activity (11) but definitive data in humans are not available. During the process of metabolic conversion of VLDL to LDL, apoB100, the main structural protein of VLDL and LDL particles, remains intact, whereas receptor-mediated uptake removes the entire particle, including apoB (18, 19). Accordingly, LDL-apoB production and clearance kinetics may be useful as a window into the upstream behavior and dynamics of apoB-containing particles and may suggest the tissue site of action of ACCi that alters plasma lipid and lipoprotein levels. In addition, LDL-apoB production rates are of interest in their own right in context of potential atherogenicity (5, 6, 7).

The half-life of VLDL-apoB is rapid (hours) while LDL-apoB exhibits a half-life of 2–5 days (20, 21, 22, 23, 24, 25). Stable isotopic metabolic tracers such as heavy water (^2^H_2_O) can be used to measure synthesis and removal rates of blood proteins, including apolipoproteins (24, 26) such as apoB100 in VLDL and LDL (25). In humans, ^2^H-label in body water rapidly equilibrates throughout all tissues and ^2^H-label rapidly enters free nonessential amino acids during intermediary metabolic processes, but not into peptide-bound amino acids (26), thereby allowing newly synthesized proteins to be labeled and measured.

Here, as part of studies to measure the effects of ACCi treatment on hepatic DNL (12), NASH patients were given heavy water before and after experimental treatment with the ACCi, firsocostat. We measured the kinetics of LDL-apoB in plasma to explore the underlying metabolic mechanisms associated with reported hypertriglyceridemia and changes of apoB particle number in ACCi-treated patients (8, 10, 11, 12, 14, 16). The primary questions were whether ACCi treatment alters the total production rate and/or the replacement rate constant (clearance) of apoB-containing particles and whether stage of liver disease influences the apoB kinetic response to ACCi treatment. In addition, we evaluated the preventive effects of concurrent therapy with the lipid-lowering agent fenofibrate and firsocostat on plasma TG concentrations and apoB kinetics.

## MATERIALS AND METHODS

### Reagents

Hyclone molecular grade water was obtained from GE health care. Sodium chloride, formic acid, acetonitrile, and methanol were obtained from Thermo Fisher Scientific. Tris base buffer, ethylenediaminetetraacetic acid, acetic acid, ammonium bicarbonate, tris(2-carboxyethyl) phosphine, iodoacetamide, and proteomics grade trypsin were obtained from Sigma-Aldrich.

### Patient treatment, characteristics, and clinical measurements

Adults 18–75 years of age with suspected NASH were studied in a phase 2a clinical trial of the ACCi, firsocostat, and fenofibrate (ClinicalTrials.gov Identifier: NCT02781584). All NASH subjects (N = 20) were administered 20 mg of firsocostat orally once daily for 12 weeks (12, 14). Of the 20 NASH subjects, 10 had F2-F3 fibrosis and 10 had cirrhosis (F4). The noncirrhotic NASH subjects treated with firsocostat subjects were enrolled with noninvasive tests using the following parameters: Screening FibroTest® < 0.75, unless a historical liver biopsy within 12 months of screening does not reveal cirrhosis, MRI-estimated proton density fat fraction with ≥ 10% steatosis, magnetic resonance elastography (MRE) with liver stiffness ≥2.88 kPa, or historical liver biopsy within 12 months of screening consistent with NASH (defined as the presence of steatosis, inflammation, and ballooning) and with stage 2–3 fibrosis according to the NASH Clinical Research Network classification (or equivalent). For cirrhotic NASH subjects treated with firsocostat, patients must have a clinical diagnosis of NAFLD and have at least one of the following criteria (a–d): a) Screening MRE with liver stiffness ≥4.67 kPa, b) A historical FibroScan® ≥ 14 kPa within 6 months of Screening, c) Screening FibroTest® ≥ 0.75, and d) A historical liver biopsy consistent with stage 4 fibrosis according to the NASH Clinical Research Network classification (or equivalent). Additional details of patient clinical characteristics have been also described elsewhere (12, 14). For the cohort of NASH subjects treated with fenofibrate and firsocostat combination therapy, all subjects had hypertriglyceridemia (TG > 150 and < 500 mg/dl) and advanced fibrosis (F3-F4) due to NASH, as determined by historic liver biopsy or liver stiffness by MRE ≥ 3.64 kPa or transient elastography (FibroScan; Echosens, Paris, France) ≥ 9.9 kPa (12). A historical liver biopsy was conducted within 6 months of screening consistent with NASH and bridging fibrosis (F3) or within 12 months of screening consistent with NASH and compensated cirrhosis (F4) in the opinion of the investigator. All patients were either pretreated with a low (48 mg) or high dose (145 mg) of fenofibrate once daily for two weeks, then a combination of firsocostat 20 mg daily plus fenofibrate at 48 mg/day (N = 14) or 145 mg/day (N = 15) for 24 weeks (supplemental Fig. S3*A*).

### Heavy water labeling protocol and measurements

Heavy water labeling was performed as part of labeling studies to investigate hepatic DNL and fibrogenesis (12). Plasma samples were taken at day 3 (baseline) and again during week 11 of treatment for LDL-apoB kinetics. ^2^H_2_O was administered for seven days in each of the labeling periods, with subjects drinking 50 ml of 70% ^2^H_2_O three times daily. During each labeling period, average body ^2^H_2_O enrichments rose to ∼0.01 fractional enrichment (1%) (see supplemental Fig. S1), as previously described (12). Blood samples were drawn after 12 hours of overnight fasting. Heavy water enrichments in each subject were analyzed by distillation followed by acetone exchange and measured via gas chromatography mass spectrometry (26).

### Sample preparation

Lipoproteins were isolated via preparative ultracentrifugation (27). NativePAGE™ Novex® Bis-Tris Gels using XCell™ SureLock™ Mini-Cell from Life Technologies was employed to further purify LDL-apoB100 from other apoB100-containing lipoproteins. The LDL-apoB100 band was excised, subjected to an in gel tryptic digest, and desalted using a C-18 SPEC tip prior to submission for mass spectrometry kinetic analysis (Thermo Fisher Scientific, In-gel Tryptic Digestion Kit).

### Serum TG and apoB measurements

Serum metabolic markers including TGs, total cholesterol, LDL-C, HDL-C, and total apoB were measured through a central laboratory (Covance, Indianapolis, IN).

### Mass spectrometry and mass isotopomer distribution analysis for calculation of LDL-apoB kinetics

LDL-apoB100 kinetics were analyzed in plasma samples obtained from subjects after 3 days of ^2^H_2_O labeling at baseline and at week 12 of ACCi treatment or fenofibrate + ACCi. LC-MS/MS was performed, as previously described, to obtain fractional replacement rates of apoB100 (26). Briefly, tryptic peptides from apoB100 were analyzed in data-dependent MS/MS mode for peptide identification and in MS mode for peptide isotopomer analysis on an Agilent 6550 ion funnel quadrupole time-of-flight mass analyzer coupled with HPLC-Chip/MS interface. Acquired MS/MS spectra were extracted and searched against the UniProtKB/Swiss-Prot human protein database (20,265 proteins, UniProt.org, release 2013_05) using Spectrum Mill Proteomics Workbench Rev B.06.00.203 software (Agilent Technologies, https://proteomics.broadinstitute.org/millhome.htm). Peptide filtering criteria included: baseline abundance of 30,000 counts, ± 5% of the predicted isotopomer, and false discovery rate of 1%. Peptide sequences provided information of elemental composition. A filtered list of apoB100 peptides was collapsed into a nonredundant peptide formula database containing peptide elemental composition, mass, and retention time. This database was used to extract mass isotopomer abundances (M0-M3) of multiple apoB100 peptides from MS-only acquisition files with the Find-by-Formula algorithm in MassHunter Qualitative Analysis software (Rev B.07.00, Agilent Technologies, https://www.agilent.com/en-us/support/software-informatics/masshunter-workstation-software/masshunter-workstation/masshunter-qualitative-analysis-b-07-00-service-pack-%28sp2%29). Isotopic enrichment was calculated as a metric of ^2^H-label incorporation from the isotopomer abundances of the peptides quantified by LC-MS, expressed as change in fractional abundance (relative intensity) of the monoisotopic peak compared to natural abundance. This is termed as excess M0 or EM0 (26). Mass Isotopomer Distribution Analysis was used to establish the isotopomer distribution pattern and enrichments in each peptide of newly synthesized apoB100. This calculation is as described previously (26) and incorporates the measured AUC of body ^2^H_2_O enrichments prior to each sample and the number of biosynthetically labile C-H bonds in each specific peptide. These parameters allow calculation of fractional synthesis (f), representing the proportion of LDL-apoB100 molecules present that were newly synthesized from the ratio of the peptide enrichment measured in the sample to the peptide enrichment for a newly synthesized peptide determined by Mass Isotopomer Distribution Analysis (24, 26, 28, 29). Fractional replacement rates (FRR, %/day) were then calculated as described previously (26, 28, 29).

FRR=−ln(1−f)/t

Half-lives (days) were calculated as

$$t_{1/2}=\ln\left(2\right)/FRR\text{.}$$

Absolute synthesis rates (ASR, mg/dl/day) were calculated by multiplying the FRR by plasma apoB100 concentration (mg/dl). We used the measured total plasma apoB100 concentration in this calculation because it is a more reliable metric of apoB100 pool size than LDL-apoB content, by avoiding potential variability of recovery through LDL isolation, and because over 90% of plasma apoB is in LDL (18, 19, 20, 21, 30). The data analysis was handled with Microsoft excel version 16.28 and Graphpad Prism version 9.2.0 (https://www.graphpad.com/scientific-software/prism/).

### Search parameters and acceptance criteria (MS/MS and/or peptide fingerprint data)

The software used for peak list generation was Agilent MassHunter Qualitative Analysis release version B.07.00. Spectrum Mill released version B.06.00.203 was the search engine for proteomic analysis based on MS/MS identifications. The sequence database searched for human protein identifications was Uniprot Release 2013_05 (31). Twenty thousand two hundred and sixty-five was the number of entries searched in the data base. Trypsin proteolysis was used. Two missed cleavages were permitted. Carbamidomethylation (C) was for fixed modifications. Acetylated lysine (K), oxidized methionine (M), N-terminal pyroglutamic acid (N-termQ), deamidated asparagine (N), and hydroxylated prolines (P) were for variable modifications. Twenty ppm and thirty ppm were the mass tolerance for precursor ions and fragment ions, respectively. The threshold score was 30% based on the minimum match peak intensity for accepting individual spectra. One percent global false discovery rate was determined by algorithms of the Spectrum Mill software (https://proteomics.broadinstitute.org/millhome.htm) and validated at the peptide and protein levels.

### Experimental design and statistical rationale

Data are presented as means ± SEM or SD as indicated in each figure. Statistical significance was calculated by a mixed model ANOVA with Tukey's multiple comparisons test. To establish differences in clinical measurements between healthy, noncirrhotic, and cirrhotic NASH patients in Table 1, statistical significance was evaluated by Kruskal-Wallis rank sum test or Fisher’s exact test. To address differences between noncirrhotic and cirrhotic NASH patients, statistical significance was computed by Wilcoxon rank sum test, Pearson’s Chi-squared test, or Wilcoxon rank sum exact test. Changes in synthesis rates (FRR or ASR) between groups were compared using a paired *t* test in the same subjects. Unpaired student *t* test with or without a Welch’s correction was used for specific comparisons as explicitly stated in the figures. Linear regression and Spearman nonparametric correlation analyses were implemented using GraphPad Prism version 9.2.0 for Mac (GraphPad Software, La Jolla, CA).

**Table 1:** Clinical and metabolic characteristics of healthy, noncirrhotic and cirrhotic NASH patients

Characteristic	Healthy, N = 10^a^	Noncirrhotic, N = 28^a^	Cirrhotic, N = 22^a^	*P* (3-Group comparison)^b^	*P* (Noncirrhotic vs. cirrhotic)^c^
Age, years	32 (25, 37)	59 (47, 64)	60 (52, 64)	<0.001	0.8
Male	6 (60%)	12 (43%)	8 (36%)	0.5	0.6
Nonhispanic ethnicity	0 (0%)	11 (39%)	12 (55%)	0.008	0.3
Diabetes	0 (0%)	18 (64%)	17 (77%)	<0.001	0.3
BMI, kg/m^∧^2	25.6 (23.4, 27.5)	34.3 (31.5, 36.9)	34.2 (29.9, 36.3)	<0.001	0.7
ALT, U/L	14 (11, 20)	46 (33, 82)	40 (32, 55)	<0.001	0.3
AST, U/L	15 (13, 16)	42 (28, 71)	46 (27, 56)	<0.001	>0.9
GGT, U/L	18 (12, 20)	36 (27, 70)	81 (36, 147)	<0.001	0.021
ALP, U/L	60 (48, 63)	71 (58, 86)	78 (59, 113)	0.027	0.4
Albumin, g/dL	4.60 (4.30, 4.68)	4.60 (4.40, 4.90)	4.50 (4.43, 4.68)	0.4	0.2
Platelets, x10^∧^3/uL	224 (207, 250)	261 (201, 290)	175 (150, 228)	0.008	0.003
Bilirubin, mg/dL	0.55 (0.40, 0.60)	0.49 (0.32, 0.70)	0.56 (0.45, 0.75)	0.5	0.3
Bile Acid, umol/L	NA	6 (5, 7)	11 (6, 16)	0.002	0.002
MRI-PDFF, %	NA	15 (12, 20)	10 (5, 13)	<0.001	<0.001
MRE, kPa	NA	3.21 (2.82, 3.59)	5.77 (5.00, 7.00)	<0.001	<0.001
FIB-4	NA	1.15 (0.99, 1.77)	1.92 (1.43, 2.59)	0.017	0.017
FibroSure/Fibrotest	NA	0.23 (0.16, 0.54)	0.50 (0.39, 0.66)	0.003	0.003
ELF	NA	9.56 (8.96, 9.91)	10.48 (9.73, 11.41)	<0.001	<0.001
APRI	NA	0.50 (0.30, 0.69)	0.69 (0.41, 1.00)	0.10	0.10
Hyaluronic acid, ng/mL	NA	55 (27, 98)	112 (70, 232)	0.001	0.001
PIII-NP, ng/mL	NA	9 (7, 12)	13 (10, 17)	0.017	0.017
TIMP-1, ng/mL	NA	260 (222, 306)	313 (257, 389)	0.031	0.031
Glucose, mg/dL	86 (83, 90)	116 (104, 138)	115 (100, 160)	<0.001	>0.9
HOMA-IR	NA	5 (4, 9)	8 (5, 15)	0.084	0.084
HbA1c, %	NA	6.35 (5.80, 7.03)	6.60 (5.75, 7.65)	0.4	0.4
Insulin, uIU/mL	7 (4, 11)	19 (14, 31)	30 (20, 38)	<0.001	0.038
Proinsulin, pmol/L	4 (3, 6)	14 (6, 27)	17 (9, 39)	<0.001	0.15
Triglycerides, mg/dL	98 (78, 107)	159 (133, 242)	162 (130, 218)	<0.001	0.7
HDL cholesterol, mg/dL	NA	42 (38, 50)	38 (32, 45)	0.13	0.13
NonHDL cholesterol, mg/dL	NA	138 (126, 156)	124 (113, 171)	0.4	0.4
VLDL triglycerides, mg/dL	NA	95 (86, 157)	100 (64, 134)	0.5	0.5
ApoA1, mg/dL	NA	143 (127, 162)	126 (117, 148)	0.054	0.054
ApoB, mg/dL	91 (86, 95)	100 (86, 113)	86 (77, 119)	0.5	0.4
Adiponectin, ng/mL	NA	3,284 (2,290, 4,307)	2,795 (2,120, 3,983)	0.5	0.5
Leptin, pg/mL	NA	25,770 (14,305, 39,357)	28,937 (15,939, 38,103)	0.9	0.9
Free fatty acid, mEq/L	0.25 (0.20, 0.30)	0.50 (0.38, 0.60)	0.50 (0.33, 0.68)	<0.001	0.6
Beta-hydroxybutyrate, mg/dL	0.70 (0.70, 0.78)	0.90 (0.90, 0.90)	1.10 (0.90, 1.40)	<0.001	0.027

Data are expressed median value (interquartile range) or as a percentage, n (%). To determine whether these groups differ between each other, statistical significance was evaluated by Kruskal-Wallis rank sum test, or Fisher’s exact test. To address differences between non-cirrhotic and cirrhotic NASH patients, statistical significance was calculated by Wilcoxon rank sum test, or Pearson’s Chi-squared test, or Wilcoxon rank sum exact test.

ALT, alanine amino transferase; ALP, alkaline phosphatase; APRI, AST to platelet ratio index; AST, aspartate amino transferase; ELF, enhanced liver fibrosis test; FIB-4, fibrosis-4; GGT, gamma-glutamyl transpeptidase; HbA1c, hemoglobin A1c; HOMA-IR, homeostatic model assessment for insulin resistance; MRE, magnetic resonance elastography; NASH, nonalcoholic steatohepatitis; PIII-NP, Type III procollagen peptide; PDFF, proton density fat fraction; TIMP-1, tissue inhibitor of metalloproteinase-1.

a Median (IQR); n (%).

b Kruskal-Wallis rank sum test; Fisher's exact test.

c Wilcoxon rank sum test; Pearson's Chi-squared test; Wilcoxon rank sum exact test.

### Study oversight

This study was approved by the institutional review board or independent ethics committees at all participating sites and was conducted in compliance with the Declaration of Helsinki, Good Clinical Practice guidelines, and local regulatory requirements.

## RESULTS

### Clinical and biochemical characteristics of healthy, noncirrhotic, and cirrhotic NASH patients

To establish patient population demographics of defined NASH subjects with clinical correlates of fibrosis and cirrhosis, common clinical and metabolic characteristics were evaluated and compared in healthy subjects and noncirrhotic or cirrhotic NASH patients (Table 1). NASH patients displayed common hallmarks of metabolic syndrome such as elevated plasma TGs, free fatty acids, ketone bodies, hyperglycemia, hyperinsulinemia, insulin resistance, and rates of diabetes as compared to healthy controls (*P* < 0.001). Markers of liver damage such as alanine amino transferase, aspartate amino transferase, gamma-glutamyl transpeptidase (*P* < 0.001), and alkaline phosphatase (0.027) were all elevated in NASH as compared to healthy controls. MRI-proton density fat fraction showed that hepatic liver fat was lower in cirrhotic NASH subjects than in noncirrhotic NASH subjects (*P* < 0.001). Noninvasive markers of liver cirrhosis including MRE (*P* < 0.001), Fib-4 (*P* = 0.17), FibroSure/Fibrotest (*P* = 0.003), Enhanced Liver Fibrosis test (*P* < 0.001), hyaluronic acid (*P* = 0.001), PIII-NP (*P* = 0.17), and tissue inhibitor of metalloproteinase-1 (*P* = 0.031) were all elevated in NASH patients with cirrhosis compared to noncirrhotic NASH subjects.

### ACCi treatment increases fasting plasma TGs in patients with NASH

**Fig. 1:**
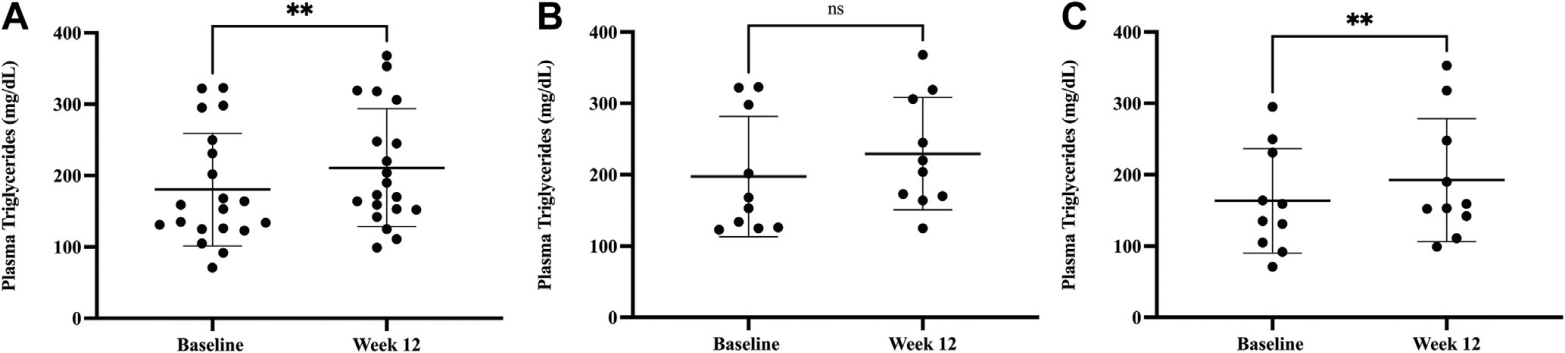
Plasma TG concentrations in NASH patients given ACCi. A: Plasma triglyceride (TG, mg/dl ± SD) concentrations in both noncirrhotic and cirrhotic NASH patients were 180 ± 79 at baseline and 211 ± 83 at week 12 of ACCi treatment. At week 12 plasma TG displayed a significant increase of 17% (*P* = 0.0056) as compared to baseline. B: Plasma triglyceride (TG, mg/dl ± SD) concentrations in non-cirrhotic NASH patients were 197.4 ± 84.4 at baseline and 229.4 ± 78.9 at week 12 of ACCi treatment (*P* = 0.1276, N = 10). C: Plasma triglyceride (TG, mg/dl ± SD) concentrations in cirrhotic NASH patients were 163.3 ± 73.3 at baseline and 192.5 ± 86.2 at week 12 of ACCi treatment (*P* = 0.0014, N = 10). Data are expressed as mean ± SD. Statistical significance was calculated by paired *t* test, ∗*P* ≤ 0.05. ACCi, acetyl-CoA carboxylase inhibitor; NASH, nonalcoholic steatohepatitis.

At baseline, plasma TG concentrations were significantly higher in noncirrhotic and cirrhotic NASH patients than in healthy subjects (*P* < 0.001), whereas apoB content did not differ (*P* = 0.5) among groups (Table 1). For the 20 patients with NASH, mean (± SD) plasma TG increased 17%, from 180 ± 79 mg/dl at baseline to 211 ± 83 mg/dl at week 12 of firsocostat treatment (*P* = 0.0056, Fig. 1A). In subgroup analysis, changes in TG were not statistically significant among the 10 noncirrhotic NASH patients (197.4 ± 84.4 at baseline vs. 229.4 ± 78.9 mg/dl at week 12, *P* = 0.1276; Fig. 1B), while significant increases were observed among the 10 cirrhotic patients (163.3 ± 73.3 at baseline vs. 192.5 ± 86.2 mg/dl at week 12, *P* = 0.0014; Fig. 1C).

### LDL-apoB synthesis rates at baseline in NASH and healthy control subjects

We measured apoB FRR in VLDL and LDL particles by ^2^H_2_O labeling combined with LC-MS/MS analysis at baseline in the different groups. The earliest time point available for analysis was at day 3 of heavy water administration. ApoB FRRs were monitored in each lipoprotein fraction after preparative ultracentrifugation. On day 3, fractional synthesis measured in VLDL-apoB in NASH subjects had reached or exceeded 100% values, which precluded inference of VLDL-apoB kinetics. LDL-apoB had an average fractional synthesis of 68% ± 19%, representing an average FRR of 37%/day or a half-life (t½) of just under 2 days. LDL-apoB FRRs were not different between healthy subjects, noncirrhotic, and cirrhotic NASH patients (supplemental Fig. S2A), which excludes a difference in LDL-apoB clearance efficiency (half-life), but LDL-apoB ASRs, calculated from plasma apoB100 concentrations multiplied by the FRR of LDL-apoB in each subject (26, 30), were significantly lower in cirrhotic versus noncirrhotic NASH subjects (*P* = 0.03, supplemental Fig. S2B).

### ACCi treatment did not affect plasma apoB concentrations in NASH patients

**Fig. 2:**
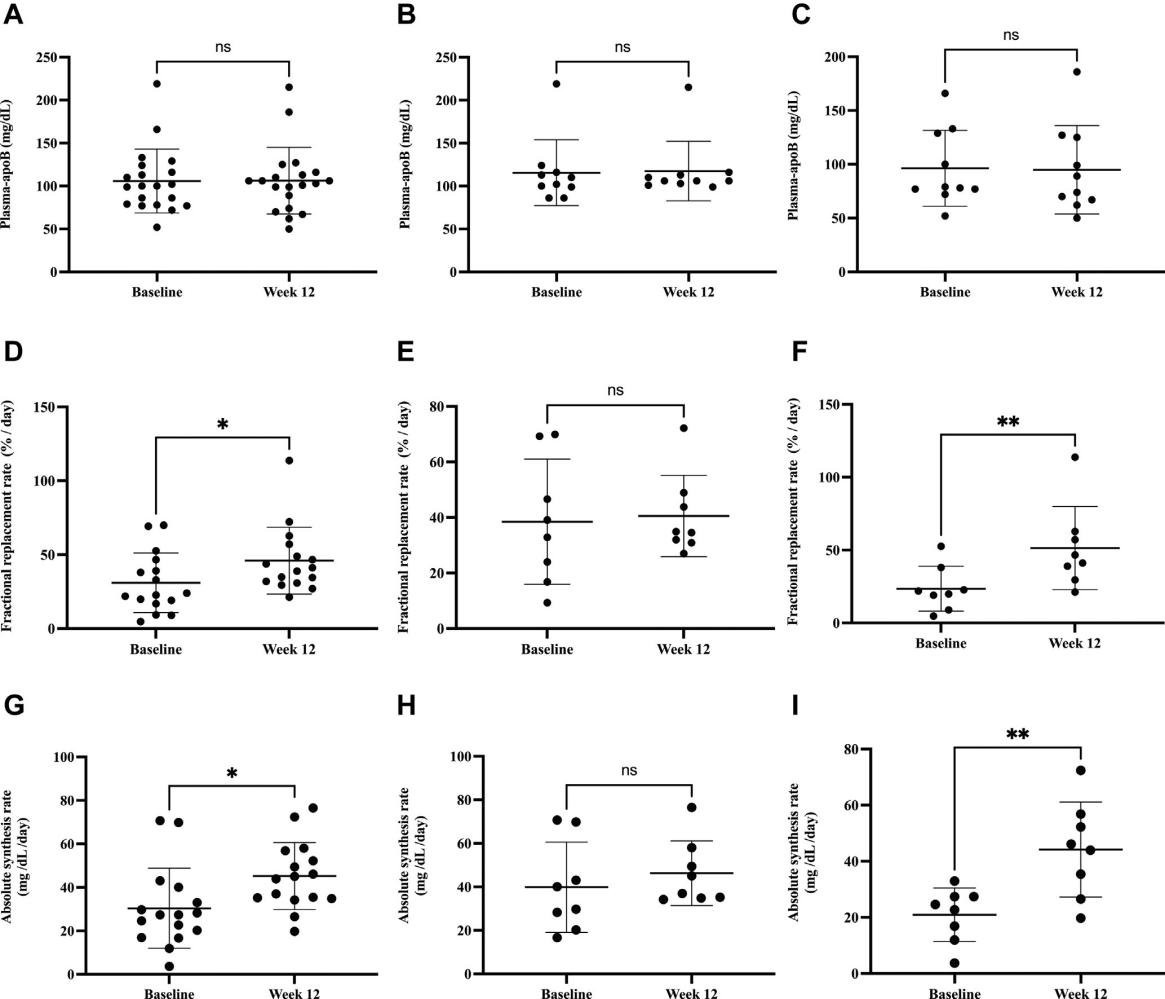
Plasma-apoB concentrations, LDL-apoB fractional replacement rates (FRR) and apoB absolute synthesis rates (ASR) in noncirrhotic and cirrhotic NASH patients at baseline and after 12 weeks of ACCi. A: Plasma apoB concentrations in combined cirrhotic and noncirrhotic subjects were 106 ± 8 and 106 ± 9 mg/dl (mean ± SD, *P* = 0.9, N = 20) at baseline and week 12, respectively. B: Noncirrhotic plasma apoB concentrations were 115.5 ± 38.4 and 117.5 ± 34.7 mg/dl (mean ± SD, *P* = 0.6461, N = 10) at baseline and week 12, respectively. C: Cirrhotic plasma apoB concentrations were 96.3 ± 35.3 and 94.9 ± 41.1 mg/dl (mean ± SD, *P* = 0.6550, N = 10) at baseline and week 12, respectively. D: LDL-apoB FRR values ± SD in combined cirrhotic and noncirrhotic subjects were 31 ± 20.2 and 46 ± 22.6%/day (*P* = 0.03, N = 16) at baseline and week 12, respectively. E: Noncirrhotic LDL-apoB FRR values ± SD were 38.5 ± 22.6 and 40.5 ± 14.6%/day at baseline and week 12, respectively, (*P* = 0.8197, N = 8). F: Cirrhotic LDL-apoB FRR values ± SD were 23.5 ± 15.4 and 51.38 ± 28.6%/day (*P* = 0.006, N = 8) at baseline and week 12, respectively. G: Plasma-apoB ASR values in combined cirrhotic and noncirrhotic subjects were 30.4 ± 18.4 and 45.2 ± 15.4 mg/dl/day (*P* = 0.016, N = 16) at baseline and week 12, respectively. H: Noncirrhotic plasma-apoB ASR values ± SD were 39.8 ± 20.8 and 46.3 ± 14.8 mg/dl/day at baseline and week 12 (*P* = 0.5060, n=8), respectively. I: Cirrhotic plasma-apoB ASR values were 21.0 ± 9.6 and 44.2 ± 17 mg/dl/day at baseline and week 12 (*P* = 0.0021, N = 8), respectively. Plasma-apoB ASR was calculated as LDL-apoB100 FRR (fraction/day) x plasma-apoB concentration (mg/dl). Data are expressed as mean values ± SD. Statistical significance was calculated by a paired *t* test, ∗*P* ≤ 0.05. ACCi, acetyl-CoA carboxylase inhibitor; ASR, absolute synthesis rate; NASH, nonalcoholic steatohepatitis.

Among patients with NASH (N = 20), mean (± SD) plasma apoB concentrations did not differ between baseline and week 12 of ACCi therapy (106 ± 8 vs. 106 ± 9 mg/dl, *P* = 0.9; Fig. 2A). Similar findings were observed in the subgroup analyses of noncirrhotic (115.5 ± 38.4 vs. 117.5 ± 34.7 mg/dl, *P* = 0.6461; Fig. 2B) and cirrhotic patients (96.3 ± 35.3 and 94.9 ± 41.1 mg/dl, *P* = 0.6550; Fig. 2C).

### ACCi treatment increases ASRs of plasma apoB in NASH patients with cirrhosis

ApoB FRRs and ASRs were measured from plasma apoB100 concentrations and the FRR of LDL-apoB in each subject. For the overall NASH population, mean (± SD) LDL-apoB FRR increased significantly from baseline to week 12 of ACCi therapy (31 ± 20.2 vs. 46 ± 22.6%/day, *P* = 0.03, N = 16, Fig. 2D), representing mean half-lives of 2.2 and 1.5 days, respectively. Subgroup analysis revealed that significant effects were restricted to NASH patients with cirrhosis. Specifically, mean (± SD) LDL-apoB FRR at baseline and week 12 of ACCi therapy were 38.5 ± 22.6%/day and 40.5 ± 14.6%/day among noncirrhotic subjects (*P* = 0.8197, N = 8, Fig. 2E), representing mean half-lives of 1.8 and 1.7 days as compared with 23.5 ± 15.4 and 51.38 ± 28.6%/day, representing mean half-lives of 2.9 and 1.4 days, respectively, in cirrhotic NASH subjects (*P* = 0.006, N = 8, Fig. 2F). Similar observations were made with respect to plasma-apoB ASR. The combined group of cirrhotic and noncirrhotic NASH patients still exhibited a significant 47% increase in plasma-apoB ASR from baseline to week 12 of ACCi therapy (30.4 ± 18.4 vs. 45.2 ± 15.4 mg/dl/day, *P* = 0.016, N = 16, Fig. 2G). Mean (± SD) plasma-apoB ASRs at baseline and week 12 of ACCi therapy were 39.8 ± 20.8 and 46.3 ± 14.8 mg/dl/day among noncirrhotic subjects (*P* = 0.51, N = 8, Fig. 2H), respectively, as compared with 21.0 ± 9.6 and 44.2 ± 17 mg/dl/day, respectively, among cirrhotic subjects (*P* = 0.002, N = 8, Fig. 2I).

### Effects of concurrent fenofibrate plus firsocostat therapy on LDL-apoB kinetics in NASH

**Fig. 3:**
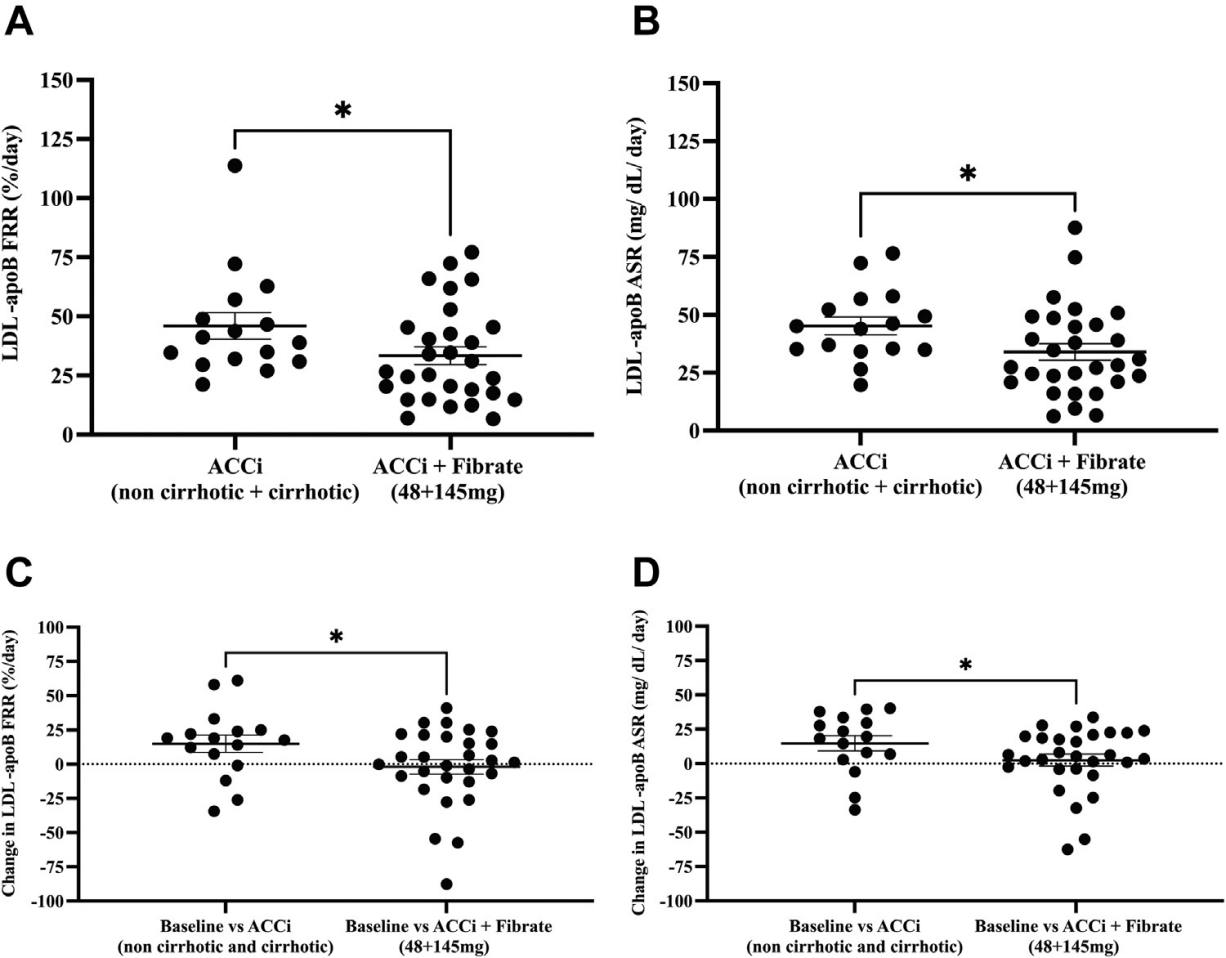
The effect of either low or high dose of fenofibrate in combination with ACCi on LDL-apoB FRR and ASR in NASH patients. A: LDL-apoB FRR (mean values ± SEM) for ACCi alone in both noncirrhotic and cirrhotic subjects versus, ACCi + two combined fenofibrate (48 mg/day and 145 mg/day) doses were 46 ± 6 and 33 ± 4%/day, respectively. ACCi+fenofibrate lowered LDL-apoB FRR than the ACCi treated group alone, *P* = 0.032. B: LDL-apoB ASR (mean values ± SEM) for ACCi alone in both noncirrhotic and cirrhotic subjects versus, ACCi + two combined fenofibrate (48 mg/day and 145 mg/day) doses were 45 ± 4 and 34 ± 4 mg/dl/day, respectively. ACCi+fenofibrate lowered LDL-apoB ASR to near baseline levels than the ACCi treated group alone, *P* = 0.026. C: Change in LDL-apoB FRR ± SEM from baseline to ACCi treatment in NASH subjects with both noncirrhosis and cirrhosis, and from baseline to ACCi + two combined fibrate doses (48 mg+145 mg) were 15 ± 6 and −2 ± 5%/day (*P* = 0.028), respectively. D: Change in LDL-apoB ASR ± SEM from baseline to ACCi treatment in NASH subjects with both noncirrhosis and cirrhosis, and from baseline to ACCi + two combined fibrate doses (48 mg+145 mg) were 15 ± 5 and 3 ± 4 mg/dl/day (*P* = 0.04), respectively. Data are expressed as mean ± SEM. Statistical significance was evaluated by one-tailed unpaired *t* test, ∗*P* ≤ 0.05. ACCi, acetyl-CoA carboxylase inhibitor; ASR, absolute synthesis rate; FRR, fractional replacement rate; NASH, nonalcoholic steatohepatitis.

We evaluated the effects of the PPAR-α agonist, fenofibrate, in combination with firsocostat on apoB kinetics in NASH subjects (supplemental Fig. S3A). Patients were pretreated with fenofibrate 48 mg/day or 145 mg/day for 2 weeks prior to adding firsocostat and LDL-apoB kinetics were sampled by heavy water labeling during the first three days of fenofibrate monotherapy. Mean (± SD) LDL-apoB FRR were 31 ± 20, 38 ± 32, and 38 ± 27%/day for the untreated, 3 days of fenofibrate 48 mg/day, and 3 days of fenofibrate 145 mg/day groups, respectively (all nonsignificant, *P* = 0.74–0.99 between each group by ANOVA, supplemental Fig. S3B). The absence of significant differences between groups suggests that three days of fenofibrate treatment did not influence acute LDL-apoB kinetics. There were no significant differences in LDL-apoB FRRs and ASRs between baseline values (after 3 days of fenofibrate treatment) and values after 12 weeks of combination therapy with fenofibrate plus the ACCi (supplemental Fig. S4A, B). ACCi in combination with both doses of fenofibrate treatment, versus ACCi alone in the mixed noncirrhotic and cirrhotic NASH patients, significantly lowered mean LDL-apoB FRR (± SEM) (33 ± 4 from 46 ± 6%/day, *P* = 0.032, Fig. 3A) and LDL-apoB ASR (± SEM) (34 ± 4 from 45 ± 4 mg/dl/day, *P* = 0.026, Fig. 3B). The change in LDL-apoB FRR ± SEM from baseline to ACCi treatment alone, and from baseline to ACCi plus fenofibrate in the two fenofibrate groups combined (48 mg/day and 145 mg/day) were 15 ± 6 and −2 ± 5%/day, respectively (*P* = 0.028, Fig. 3C). Additionally, the change in LDL-apoB ASR (mean ± SEM) from baseline to ACCi treatment alone and from baseline to ACCi plus fenofibrate in the two fenofibrate combined groups (48 mg/day and 145 mg/day) were 15 ± 5 and 3 ± 4 mg/dl/day, respectively (*P* = 0.04, Fig. 3D). Subgroup analysis of the change in LDL-apoB ASR revealed nonsignificant effects between groups (supplemental Fig. S5B), except for the change in LDL-apoB FRRs from baseline to ACCi alone in the cirrhotic group compared to baseline versus ACCi + low dose fibrate in subjects with no cirrhosis (*P* = 0.05, supplemental Fig. S5A).

### Correlation between changes in plasma TG and plasma apoB content or kinetics

Changes in plasma-apoB100 and TG levels were compared to changes in plasma apoB100 kinetics from baseline to 12 weeks of ACCi treatment. While changes in TG and plasma-apoB100 content between baseline and week 12 were significantly correlated (r = 0.47, *P* = 0.018, Table 2), no significant correlations were observed between changes in TG concentrations and plasma-apoB kinetics, with a trend in the negative direction. Interestingly, the change in LDL-apoB FRR and plasma apoB concentration from baseline to week 12 of ACCi treatment displayed a borderline significant association (*P* = 0.053) and negative correlation (r = −0.42).

**Table 2:** Correlations between the change in plasma triglycerides, plasma-apoB, LDL-apoB FRR, and LDL-apoB ASR at week 12 as compared to baseline after ACCi treatment

Correlations	Spearman r	*P*
Triglyceride concentration versus plasma-apoB concentration	0.47	a0.018
Triglyceride concentration versus LDL-apoB FRR	−0.18	0.25
Triglyceride concentration versus LDL-apoB ASR	−0.14	0.31
Plasma-apoB concentration versus LDL-apoB FRR	−0.42	0.053

Data are expressed as a spearman r correlation value with corresponding *P* value. Change in mean plasma apoB concentrations, triglyceride concentrations, and LDL-apoB FRR or LDL-apoB ASR kinetic values from baseline to week 12 were calculated by the following formula, week 12 values - baseline value.

a *P* ≤ 0.05 based on spearman correlation analysis. ACCi, acetyl-CoA carboxylase inhibitor; ASR, absolute synthesis rate; FRR, fractional replacement rate.

## DISCUSSION

Treatment with ACC inhibitors leads to hypertriglyceridemia in a subset of patients with NASH (8, 10, 11, 12, 13, 14, 15, 16). Our goals were to determine whether treatment with a liver specific ACCi, firsocostat (32), which has been reported to increase plasma TGs and variably to increase apoB concentrations, are associated with altered LDL-apoB particle production rate or half-life (clearance), whether the stage of liver disease alters the LDL-apoB kinetic response to ACCi therapy and whether concurrent treatment with a fibrate can prevent changes in LDL-apoB kinetics.

Endogenously derived TG are trafficked in the blood primarily in VLDL. During the process of metabolic conversion of VLDL to LDL, the main structural protein of these particles, apoB100, remains intact, whereas receptor-mediated uptake removes the intact particle, which includes nonexchangeable apoB100 that persists throughout the lifetime of a LDL particle (19). The export of VLDL from the liver into circulation is reliant on hepatic apoB synthesis (19). The majority (∼90%) of circulating apoB100 resides in LDL. The half-life of VLDL is hours, whereas LDL, for which VLDL is the intravascular precursor, has a half-life of days (20, 21, 22, 23, 24, 25). In this study, blood was not sampled during the first day of heavy water labeling to measure VLDL-apoB kinetics but the sample collected at 3 days of labeling enabled analysis of LDL-apoB kinetics.

We did not measure VLDL-TG or VLDL-apoB production rates, but the finding of LDL-apoB overproduction is important in its own right for at least two reasons. First, LDL-apoB production rate is of interest in terms of metabolic site of drug action and potential atherogenic risk (5, 6, 7, 33). Second, in other clinical settings such as type 2 diabetes, hypertriglyceridemia is driven primarily by hepatic overproduction of apoB-containing molecules (34, 35, 36). It is therefore reasonable to infer overproduction of apoB-containing particles from overproduction of LDL-apoB particles by the liver (24, 30), at least as a hypothesis-generating observation. The half-life of ∼2 days measured here for apoB in the LDL fraction is consistent with prior reports (20, 21, 22, 23, 24, 25).

In NASH patients in this study, plasma concentrations of TG but not apoB increased after 12 weeks of treatment with the ACCi firsocostat (Figs. 1A and 2A). Our primary finding here is the significant increase in ASR of LDL-apoB at week 12 of firsocostat treatment, restricted to the subgroup of NASH patients with cirrhosis (Fig. 2F, I). The finding that the replacement rate constant (FRR) was more rapid, not slower, argues against an LDL-apoB clearance defect induced by ACCi.

Some technical points about lipoprotein kinetics are worth noting. ASR is the product of FRR and pool size (18, 19, 20, 21, 30) and represents the biochemical production rate, expressed in units of mass per time. An increase in pool size with no slowing of half-life means that the change in pool size is due to higher production rates, not slower removal rates. Indeed, this is the main information gained from a metabolic labeling study of this type and the result here was unambiguous. Hypertriglyceridemia might have involved no change in apoB100 turnover, which would have suggested altered plasma lipid turnover without a change in particle metabolism (e.g., a lipoprotein lipase or ApoCIII effect) (11). The correlation between relative changes in plasma-apoB and TG concentrations at week 12 of firsocostat treatment suggests a relationship between increased particle production and plasma TG concentrations (Table 2). Since our study did not directly assess VLDL conversion into LDL, however, we cannot directly confirm this hypothesis from our study.

We did not explore potential molecular signals, but these observations should be considered in context of previous reports describing higher VLDL secretion in a genetic model of ACC ablation (ACC double knock-out mice) and hypertriglyceridemia in humans treated with different ACCi compounds (8, 10, 11, 12, 13, 14, 15, 16). Reduced PUFAs in the liver have been reported after ACCi treatment (8). PUFAs act as key regulators of SREBP-1C activity by inhibiting its proteolytic processing (37). PUFA reduction, specifically omega 3- and 6-containing PUFAs such as arachidonic acid and docosahexaenoic acid were suggested to activate SREBP-1c and reduce PPARα activity (8) and PUFA supplementation in the double knockout ACC mice normalized TG levels (8).

Kim *et al.* also showed upregulation of downstream genes to SREBP-1C, such as glycerol phosphate acyl transferase-1, associated with increased VLDL secretion in liver specific ACC knockout mice (8). In fasted overnight rodents treated with an ACCi after Poloxamer 405 administration to inhibit lipolytic clearance of TG rich lipoproteins, Goedeke, and colleagues reported an increase in VLDL secretion rates (11). Insulin resistance in NASH patients (1, 2, 3, 4) is associated with elevated NEFAs flux, as shown by elevated levels of NEFAs here (Table 1), providing an alternate pool of fatty acids for TG synthesis (8). Our data in humans are consistent with an effect of ACCi on hepatic TG and apoB particle production as the site of action.

Moreover, Loomba *et. al.* conducted nuclear magnetic resonance lipoprotein analysis in a similar cohort of NASH patients treated with firsocostat in a 12-weeks phase 2a study (14). While increased particle number and TG concentration of VLDL were observed over 1 week of ACCi treatment, the number of small LDL particles, total cholesterol, HDL-C, and LDL-C concentrations and particle number, along with glycemic parameters, did not change during the study in comparison to placebo (14). In a multivariate analysis adjusting for demographics and lipids at baseline, grade 3 or 4 hypertriglyceridemia (>500 mg/dl) during ACCi treatment was associated with patients whose baseline plasma TG levels were over 250 mg/dl. Importantly, despite ongoing treatment with firsocostat, treatment with fibrates or fish oil led to resolution of grade 3 or 4 hypertriglyceridemia (14). The utility of fibrates to mitigate ACCi-induced hypertriglyceridemia has been evaluated in several clinical studies in addition to studies in rodents (11, 13, 15). In a proof-of-concept study of NASH patients with hypertriglyceridemia (>150 mg/dl) and advanced (F3-F4) fibrosis, Lawitz *et al.* showed that a 2-weeks course of preemptive therapy with fenofibrate (48 or 145 mg) prevented any increase in TG after 24 weeks of fenofibrate and firsocostat combination therapy (13). They also confirmed that fenofibrate (145 mg) prevents TG elevations in the setting of combination therapy with firsocostat and the farnesoid X receptor agonist, cilofexor, in hypertriglyceridemic patients with NASH (13). These data suggest that concurrent ACCi and fenofibrate treatment may prevent the increase in plasma TG with ACCi treatment alone.

To explore these clinical observations, we evaluated whether combination therapy with fenofibrate and firsocostat also altered LDL-apoB production rates. When the data for both doses of fenofibrate with ACCi treatment and the stages of NASH were combined, apoB FRR and ASR were significantly lower for combined treatment than for ACCi treatment alone in the cross-sectional analysis (Fig. 3A, B), although the change in LDL-apoB kinetics in the low or high fibrate doses + ACCi treatments were not significantly different from ACCi treatment alone in either subgroup classified by cirrhosis state alone (supplemental Fig. S5A, B). Our longitudinal analyses were potentially confounded by the initial 3 days of fibrate therapy at baseline but longitudinal comparisons supported the cross-sectional analyses. There were no apparent effects of the initial 3 days of fibrate treatment on apoB FRR (supplemental Fig. S3B) and the addition of ACCi treatment did not increase apoB FRR (supplemental Fig. S4A, B). Moreover, we observed a significant reduction in the change in LDL-apoB FRR and ASR with the ACCi + two combined fibrate doses (Fig. 3C, D). Previous lipoprotein kinetic studies have been reported in men with metabolic syndrome treated with fibrates alone. In one study, treatment with fenofibrate 200 mg/day for 5 weeks led to increased fractional catabolic rate and decreased pool size of apoB-containing particles (38). Caslake *et. al.* reported decreased VLDL particle size after fenofibrate treatment, as well as increased LDL-apoB degradation by the receptor route but not for receptor independent routes (39). Lastly, fenofibrate treatment lowered VLDL-apoB concentrations and secretion rates in NAFLD patients, consistent with our data showing suppressed LDL-apoB ASR with concurrent fenofibrate and ACCi treatment (Fig. 3B, (40)).

We observed that NASH patients with cirrhosis have lower apoB ASR than noncirrhotic and healthy counterparts (supplemental Fig. S2B). Both noncirrhotic NASH and cirrhotic NASH patients were insulin resistant as indicated by homeostatic model assessment for insulin resistance but the cirrhotic NASH displayed higher levels of circulating insulin (Table 1). Differences in insulin signaling could potentially explain alterations in basal apoB secretion rates (34, 35, 36, 41). The cirrhotic NASH patents also showed lower hepatic fat than noncirrhotic NASH patients (Table 1 and supplemental Fig. S2B), which might lower apoB secretion (5, 6, 7, 41). Further studies are warranted to understand these differences in LDL-apoB metabolism.

Importantly, we observed different effects of the ACCi on lipid and lipoprotein metabolism between noncirrhotic and cirrhotic NASH patients (12, 14). After ACCi treatment, cirrhotic NASH patients displayed elevated plasma TG levels and LDL-apoB FRR and ASR as compared to noncirrhotic subjects (Figs. 1C and 2F, I). The explanation for these differential effects remains uncertain. These data could indicate some restoration of hepatocellular function with ACCi treatment in cirrhosis (42). Data to support this hypothesis include improvements in liver function, including decreased liver fat content, liver stiffness, neuroinflammatory activity, and fibrosis (11, 12, 13, 14, 15, 42). In rodents treated with ACCi’s, decreased expression of markers of macrophage activation and fibrosis such as C-C motif chemokine 2 and collagen alpha-1(I) chain were observed (42). Lower staining of α-smooth muscle actin, and cluster of differentiation 3, markers of hepatic stellate cell activation, as well as fibrogenesis, and T cell activation have been reported (16, 42).

In conclusion, we show here that treatment with the ACCi firsocostat significantly increases the synthesis rate of apoB-containing LDL particles in NASH subjects with cirrhosis, without a significant increase in plasma apoB concentrations (Graphical abstract). These results suggest that the site of action of previously reported effects of ACCi treatment on plasma TG concentrations is the liver. Fenofibrate combination therapy prevented the increased LDL-apoB particle production induced by firsocostat therapy.

## Data availability

All data can be viewed in the manuscript. Any or additional data is available upon reviewer’s request from the corresponding author.

## Supplemental data

This article contains supplemental data.

## Conflict of interest

The authors declare the following financial interests/personal relationships which may be considered as potential competing interests.

## Supplemental data

Data filter 1

Data filter 2

Data filter 3

Data filter 4

Data filter 5

Data filter 6

Data filter 7

Data filter 8

Data filter 9

Data filter 10

Data filter 11

Summary table 1

Summary table 2

Summary table 3

Summary table 4

Summary table 5

Summary table 6

Summary table 7

Summary table 8

Summary table 9

Summary table 10

Summary table 11
