# Supplementary material for: Acetyl-CoA carboxylase inhibitor increases LDL-apoB production rate in NASH with cirrhosis: prevention by fenofibrate
Source: J Lipid Res. 2023 Feb 2;64(3):100339. doi: 10.1016/j.jlr.2023.100339 (PMC10017426; doi:10.1016/j.jlr.2023.100339)
Supplement: Supplemental materials [file mmc23.pdf]

SUPPLEMENTAL INFORMATION:

Acetyl-CoA Carboxylase Inhibitor Increases LDL-apoB Production Rate in NASH with Cirrhosis: Prevention by Fenofibrate.

[1]Mohamad Dandan, [1]Julia Han, [1]Sabrina Mann, [1]Rachel Kim, [1]Kelvin Li, [1]Hussein Mohammed, [2]Jen-Chieh Chuang, [2]Kaiyi Zhu, [2]Andrew N. Billin, [2]Ryan S. Huss, [2]Chuhan Chung, [2]Robert P. Myers, [1,3]Marc Hellerstein.

[1]Department of Nutritional Sciences and Toxicology, Graduate Program in Metabolic Biology, University of California at Berkeley, Berkeley, California, USA.

[2]Gilead Sciences, Inc, Foster City, California, USA.

[3]To whom correspondence should be addressed: Marc Hellerstein, Professor of Nutritional Sciences and Toxicology, Department of Nutritional Sciences and Toxicology, University of California at Berkeley, Berkeley, California, USA. 309 Morgan Hall, Berkeley, California, 94720, 510-642-0646, march@berkeley.edu

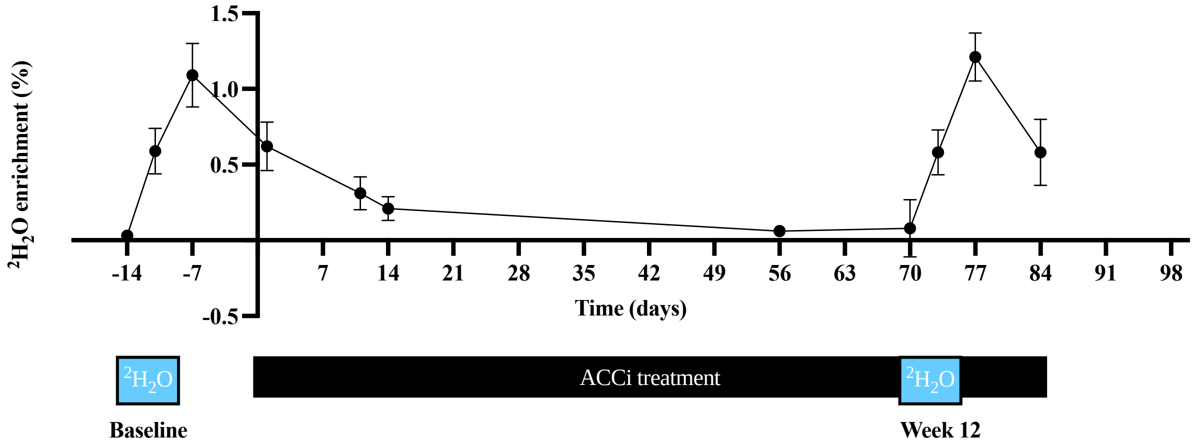

Supplemental figure S1: Heavy water labeling study design. Labeling design and time course of ^2H_2O enrichment over the study period with average body water enrichments of both non-cirrhotic and cirrhotic NASH subjects (n=10-20 per time point). Subjects were labeled with heavy water at baseline and week 12 of ACCi treatment. At day 0, subjects were administered firsocostat 20 mg per day orally once a day, for 12 weeks.

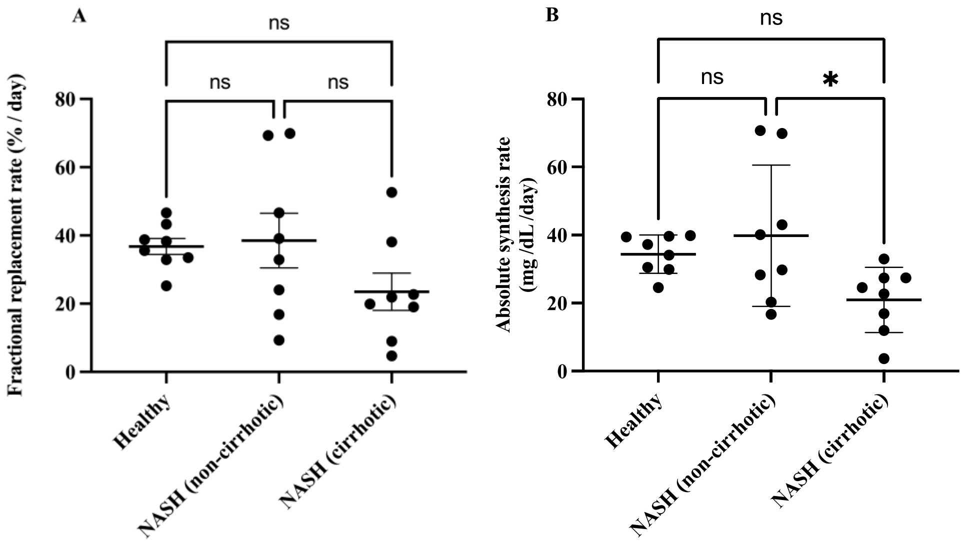

Supplemental figure S2: Baseline characteristics of LDL-apoB fractional replacement rates (FRR), and apoB absolute synthesis rates (ASR) in healthy volunteers vs baseline values from non-cirrhotic or cirrhotic NASH patients treated with ACCi. **A)** LDL-apoB FRR (%/day) in non-cirrhotic (n=8, p=0.98) and cirrhotic NASH patients (n=8, p=0.25) were not significantly different when compared to healthy volunteers (n=8). **B)** LDL-apoB ASR (mg/dL/day) in non-cirrhotic (n=8, p=0.71) and cirrhotic NASH patients (n=8, p=0.14) were not significantly different when compared to healthy volunteers (n=8), but LDL-apoB ASRs were significantly lower in cirrhotic (p=0.03) than non-cirrhotic NASH patients. Data are expressed as mean ± SEM. Statistical significance was evaluated by an ANOVA, *p≤0.05.

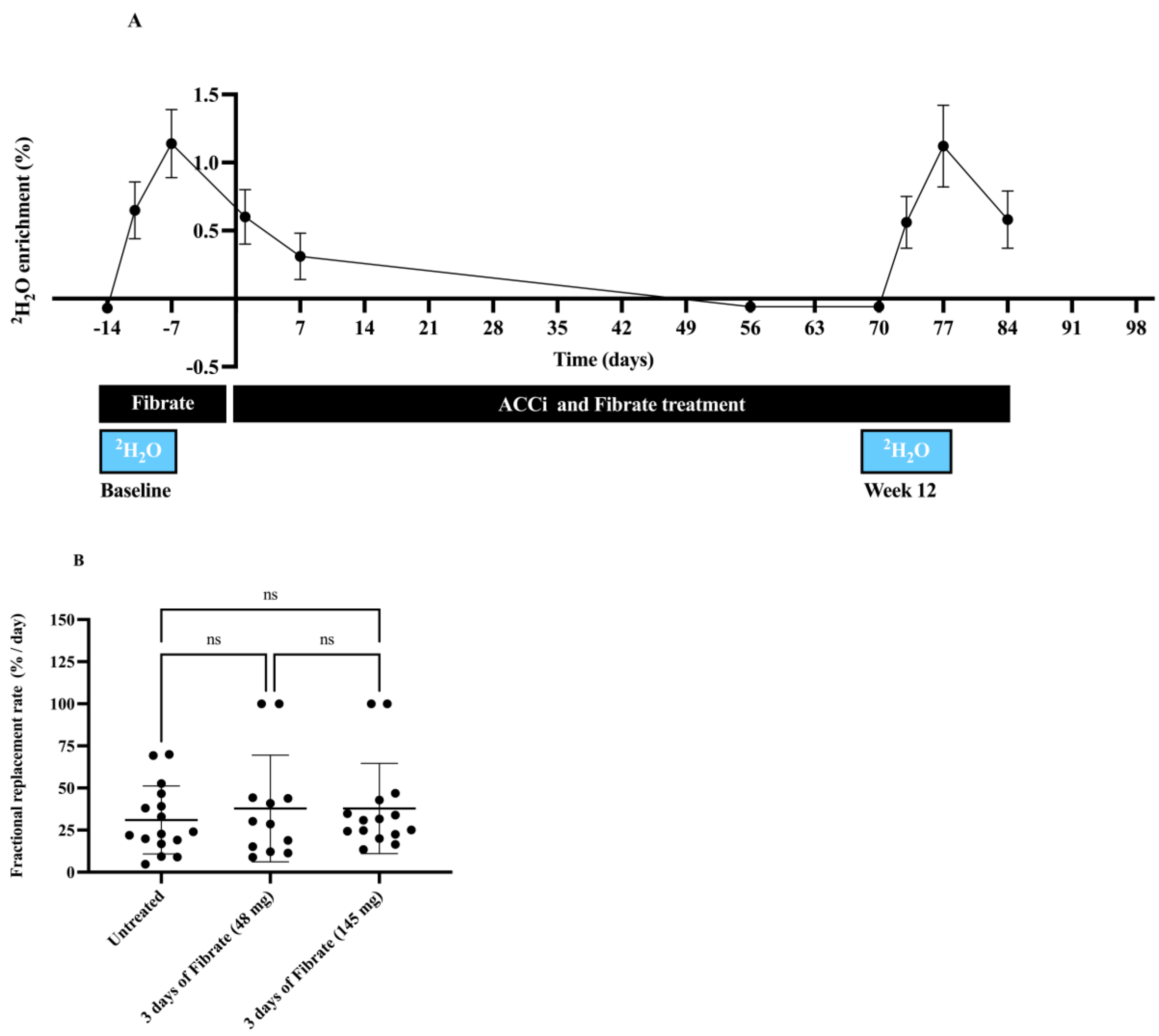

Supplemental figure S3: Lack of effect of 3 days of fenofibrate administration at baseline on apoB FRR. **A)** Study design; **B)** LDL-apoB FRR (%/day). No significant differences were observed between treatments. Data are expressed as mean ± SD. Statistical significance was calculated by an ANOVA, *p≤0.05.

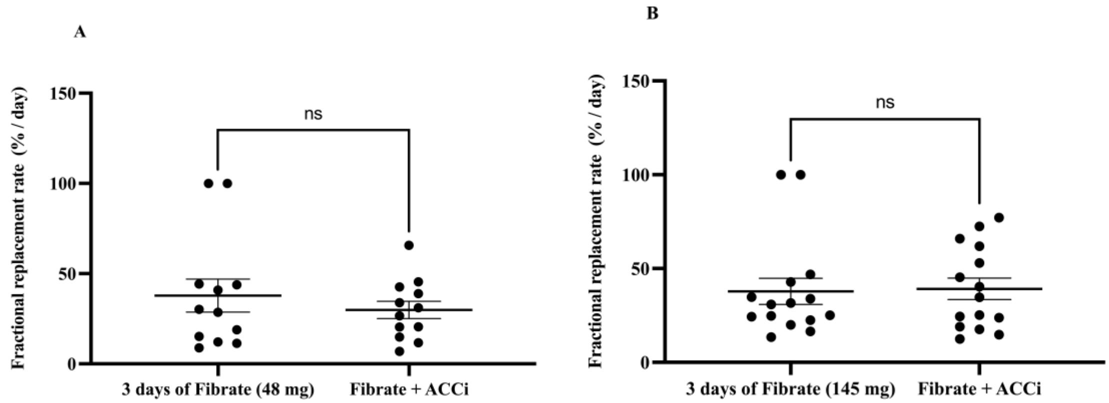

Supplemental figure S4: Effects of 12 weeks of ACCi + fenofibrate therapy on apoB FRR (%/day).
A) ACCi + Fenofibrate 48 mg/day; **B)** ACCi + Fenofibrate 145 mg/day. No difference was observed between treatments. Data are expressed as mean ± SEM. Statistical significance was evaluated by a paired T. test, $*p \leq 0.05$.

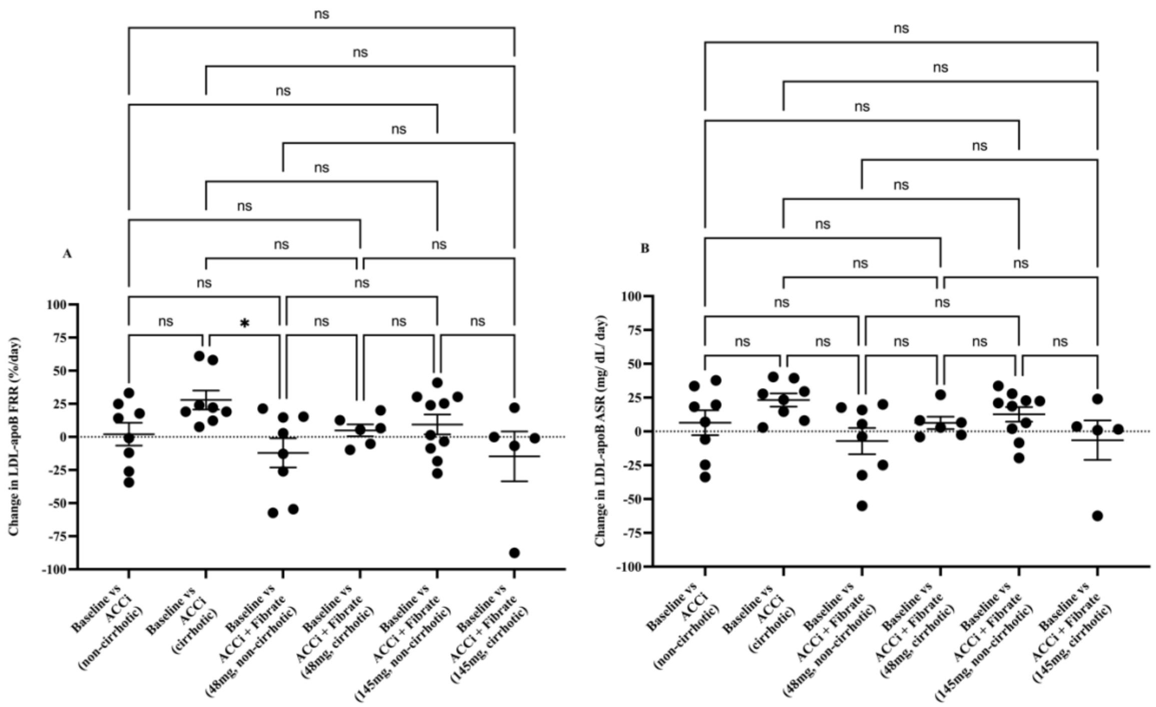

Supplemental figure S5: The effect of either low or high dose of fenofibrate in combination with ACCi on LDL-apoB FRR in NASH patients with or without cirrhosis, expressed as changes from baseline. **A)** Change in LDL-apoB FRR (mean ± SEM) from baseline to ACCi treatment in NASH subjects with non-cirrhosis or cirrhosis, and from baseline to ACCi + two individual low or high fibrate doses (48mg or 145mg) with or without cirrhosis were 2 ± 9, 28 ± 7, -12 ± 11, 5 ± 5, 9 ± 7, -15 ± 19, respectively. No statistical significance was observed between each group, except for baseline vs ACCi cirrhotic groups, as compared to baseline vs ACCi + low dose fibrate in subjects without cirrhosis (p=0.05). **B)** Change in LDL-apoB ASR (mean ± SEM) from baseline to ACCi treatment in NASH subjects with non-cirrhosis or cirrhosis, and from baseline to ACCi + two individual low or high fibrate doses (48mg or 145mg) with or without cirrhosis were 6 ± 9, 23 ± 5, -7 ± 10, 6 ± 5, 13 ± 5, -7 ± 15, respectively. Data are expressed as mean ± SEM. Statistical significance between each group was calculated by ANOVA, *p≤0.05.

Additional files

The summary table and data filter excel files are provided to download. All necessary mass isotopomer distribution data are shown in the summary table files. The summary table data is filtered as a data filtered file for computation of the fractional synthesis based on algorithms of the Hellerstein lab. For each excel data filter file, the parameters tab provides the different parameters used for data filtering that include retention times, base peak abundance, root mean score error filter, theoretical upper and lower limit for EM0, standard deviation between peptides, and isotopomers standard deviations, etc.
